# Tetra-μ-benzoato-κ^4^ *O*:*O*′;κ^3^ *O*:*O*,*O*′;κ^3^ *O*,*O*′:*O*′-bis­[(benzoato-κ^2^ *O*,*O*′)(1,10-phenanthroline-κ^2^ *N*,*N*′)neodymium(III)]

**DOI:** 10.1107/S1600536810003041

**Published:** 2010-01-30

**Authors:** Ping Howe Ooi, Siang Guan Teoh, Jia Hao Goh, Hoong-Kun Fun

**Affiliations:** a School of Chemical Sciences, Universiti Sains Malaysia, 11800 USM, Penang, Malaysia; b X-ray Crystallography Unit, School of Physics, Universiti Sains Malaysia, 11800 USM, Penang, Malaysia

## Abstract

The complete mol­ecule of the title compound, [Nd_2_(C_7_H_5_O_2_)_6_(C_12_H_8_N_2_)_2_], is generated by a crystallographic inversion center. The two Nd^III^ ions are linked by four bridging benzoate ions, with an Nd⋯Nd separation of 4.0360 (2) Å. As well as the bridging ligands, each Nd^III^ ion is coordinated by one *N*,*N*′-bidentate phenanthroline ligand and an *O*,*O*′-bidentate benzoate ion. The resulting irregular nine-coordinated geometry of the Nd^III^ ion is completed by seven O and two N atoms. The mol­ecular structure is stabilized by intra­molecular C—H⋯O hydrogen bonds. In the crystal structure, mol­ecules are linked into infinite chains along the *c* axis by inter­molecular C—H⋯O hydrogen bonds. The crystal structure is consolidated by weak inter­molecular C—H⋯π inter­actions.

## Related literature

For general background to and applications of Nd^III^ complexes, see: Swavey & Swavey (2009 ▶). For related Ln–benzoato complexes, see: Niu *et al.* (1999 ▶); Niu *et al.* (2002 ▶); Shi *et al.* (2001 ▶). For the stability of the temperature controller used for the data collection, see: Cosier & Glazer (1986 ▶).
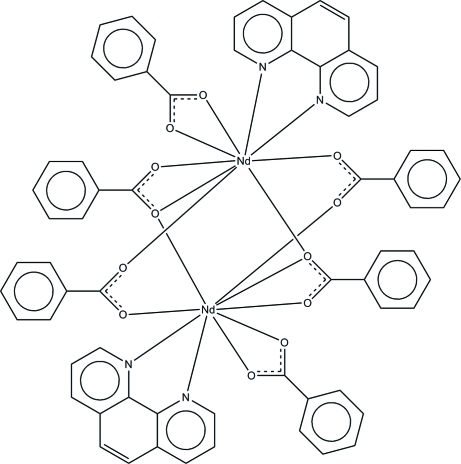


## Experimental

### 

#### Crystal data

[Nd_2_(C_7_H_5_O_2_)_6_(C_12_H_8_N_2_)_2_]
*M* *_r_* = 1375.55
Triclinic, 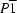

*a* = 10.7954 (3) Å
*b* = 11.8702 (4) Å
*c* = 12.2660 (7) Å
α = 104.925 (1)°
β = 93.831 (1)°
γ = 112.877 (1)°
*V* = 1374.49 (10) Å^3^
*Z* = 1
Mo *K*α radiation
μ = 1.94 mm^−1^
*T* = 100 K
0.69 × 0.41 × 0.13 mm

#### Data collection

Bruker SMART APEX DUO CCD diffractometer
Absorption correction: multi-scan *SADABS* (Bruker, 2009 ▶) *T*_min_ = 0.347, *T*_max_ = 0.784
46897 measured reflections
11933 independent reflections
11529 reflections with *I* > 2σ(*I*)
*R*_int_ = 0.020

#### Refinement

*R*[*F* ^2^ > 2σ(*F* ^2^)] = 0.016
*wR*(*F* ^2^) = 0.071
*S* = 1.39
11933 reflections
379 parameters
H-atom parameters constrained
Δρ_max_ = 1.26 e Å^−3^
Δρ_min_ = −1.51 e Å^−3^

### 

Data collection: *APEX2* (Bruker, 2009 ▶); cell refinement: *SAINT* (Bruker, 2009 ▶); data reduction: *SAINT*; program(s) used to solve structure: *SHELXTL* (Sheldrick, 2008 ▶); program(s) used to refine structure: *SHELXTL*; molecular graphics: *SHELXTL*; software used to prepare material for publication: *SHELXTL* and *PLATON* (Spek, 2009 ▶).

## Supplementary Material

Crystal structure: contains datablocks global, I. DOI: 10.1107/S1600536810003041/hb5308sup1.cif

Structure factors: contains datablocks I. DOI: 10.1107/S1600536810003041/hb5308Isup2.hkl

Additional supplementary materials:  crystallographic information; 3D view; checkCIF report

## Figures and Tables

**Table 1:** Selected bond lengths (Å)

Nd1—O4^i^	2.3856 (10)
Nd1—O6	2.4060 (10)
Nd1—O5^i^	2.4230 (10)
Nd1—O2	2.4600 (10)
Nd1—O3	2.4810 (10)
Nd1—O1	2.5475 (10)
Nd1—N1	2.6288 (12)
Nd1—N2	2.6870 (11)
Nd1—O4	2.8039 (10)

Symmetry code: (i) 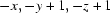.

**Table 2:** Hydrogen-bond geometry (Å, °) *Cg*1, *Cg*2 and *Cg*5 are the centroids of the C28–C33, C21–C26 and C14–C19 phenyl rings, respectively. *Cg*3 and *Cg*4 are the centroids of the N2/C8–C12 and N1/C1–C15 pyridine rings, respectively.

*D*—H⋯*A*	*D*—H	H⋯*A*	*D*⋯*A*	*D*—H⋯*A*
C3—H3*A*⋯O1^ii^	0.93	2.57	3.4729 (19)	163
C11—H11*A*⋯O6	0.93	2.50	3.1239 (19)	125
C26—H26*A*⋯O2^i^	0.93	2.56	3.4393 (19)	158
C7—H7*A*⋯*Cg*1^iii^	0.93	2.89	3.4554 (18)	121
C16—H16*A*⋯*Cg*2^iv^	0.93	2.98	3.7544 (18)	141
C17—H17*A*⋯*Cg*3^v^	0.93	2.94	3.7287 (19)	143
C24—H24*A*⋯*Cg*4^vi^	0.93	2.81	3.6620 (17)	153
C30—H30*A*⋯*Cg*5^vii^	0.93	2.73	3.6435 (18)	167

Symmetry codes: (i) 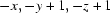; (ii) 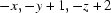; (iii) 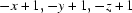; (iv) 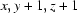; (v) 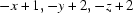; (vi) 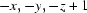; (vii) 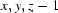.

**Fig. 1.**
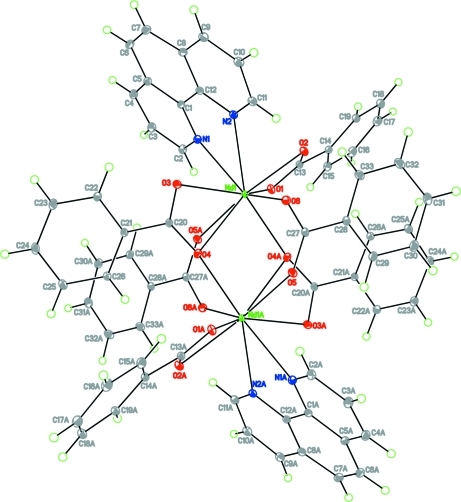
The molecular structure of (I) showing 20% probability displacement ellipsoids for non-H atoms. The suffix A corresponds to the symmetry code [-x, -y+1, -z+1].

**Fig. 2.**
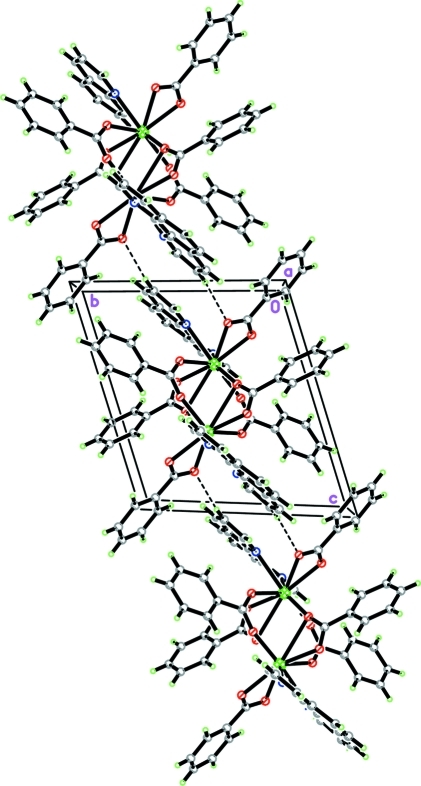
The crystal structure of (I), viewed along the a axis, showing one-dimensional infinite chains along the c axis. Intermolecular hydrogen bonds are shown as dashed lines.

## supplementary crystallographic information

### Comment

Lanthanide complexes with organic ligands, especially Nd^III^ complexes, are often used in magnetic resonance imaging (MRI) because Nd^III^ complexes emit in the near infrared region (NIR) (Swavey & Swavey, 2009). The crystal structure obtained from this complex are slightly different from the other Ln-benzoato complexes, such as La^III^ (Shi *et al.*, 2001), Sm^III^ (Niu *et al.*, 1999), and Gd^III^ (Niu *et al.*, 2002) due to the lanthanide contraction.

The asymmetric unit of the title complex (Fig. 1) lies on a crystallographic inversion center and comprises of one-half molecule [symmetry code of atoms labelled with suffix A: -x, -y+1, -z+1]. The two Nd^III^ ions are linked by four benzoate ions, with an Nd—Nd distance of 4.0360 (2) Å. Among the four benzoate ions, two of them also behave as chelating ligands to the Nd^III^ ions. Each Nd^III^ ion is coordinated by one phenanthroline heterocycle and a bidentate benzoate ion. The irregular nine-coordinated geometry of the Nd^III^ ion is completed by seven benzoate O atoms and two phenanthroline N atoms. Intramolecular C11—H11A···O6 and C26—H26A···O2 hydrogen bonds (Table 2) stabilize the molecular structure. Bond lengths of Nd—O and Nd—N are listed in Table 1. All other bond lengths and angles are comparable to a closely La-benzoato complex (Shi *et al.*, 2001).

In the crystal structure, intermolecular C3—H3A···O1 hydrogen bonds (Table 2) link the molecules into infinite chains along the *c* axis (Fig. 2). The crystal structure is further stabilized by weak intermolecular C7A—H7A···*Cg*1, C16—H16A···*Cg*2, C17—H17A···*Cg*3, C24—H24A···*Cg*4 and C30—H30A···*Cg*5 interactions (Table 2).

### Experimental

NdCl_3_.6H_2_O was prepared by dissolving neodymium oxide in hydrochloric acid and then dried. Metal salt (0.5 mmol) in methanol was added into a solution (methanol-H_2_O, 1:1) of 1,10-phenanthroline (0.5 mmol) and benzoic acid (1.5 mmol). The mixture was refluxed for 24 h to give a pink solution. The solution was filtered at room temperature and purple plates of (I) were obtained after 10 days.

### Refinement

All aromatic hydrogen atoms were placed in their calculated positions, with C—H = 0.93 Å, and refined using a riding model with *U*_iso_ = 1.2 *U*_eq_(C). The highest residual electron density peak is located at 1.25 Å from O4 and the deepest hole is located at 1.51 Å from Nd1.

### Crystal data

**Table:**

[Nd_2_(C_7_H_5_O_2_)_6_(C_12_H_8_N_2_)_2_]	*Z* = 1
*M**_r_* = 1375.55	*F*(000) = 686
Triclinic, *P*1	*D*_x_ = 1.662 Mg m^−^^3^
Hall symbol: -P 1	Mo *K*α radiation, λ = 0.71073 Å
*a* = 10.7954 (3) Å	Cell parameters from 9761 reflections
*b* = 11.8702 (4) Å	θ = 3.0–37.6°
*c* = 12.2660 (7) Å	µ = 1.94 mm^−^^1^
α = 104.925 (1)°	*T* = 100 K
β = 93.831 (1)°	Plate, purple
γ = 112.877 (1)°	0.69 × 0.41 × 0.13 mm
*V* = 1374.49 (10) Å^3^	

### Data collection

**Table:**

Bruker SMART APEX DUO CCD diffractometer	11933 independent reflections
Radiation source: fine-focus sealed tube	11529 reflections with *I* > 2σ(*I*)
graphite	*R*_int_ = 0.020
φ and ω scans	θ_max_ = 35.0°, θ_min_ = 2.7°
Absorption correction: multi-scan *SADABS* (Bruker, 2009)	*h* = −17→17
*T*_min_ = 0.347, *T*_max_ = 0.784	*k* = −18→19
46897 measured reflections	*l* = −19→19

### Refinement

**Table:**

Refinement on *F*^2^	Primary atom site location: structure-invariant direct methods
Least-squares matrix: full	Secondary atom site location: difference Fourier map
*R*[*F*^2^ > 2σ(*F*^2^)] = 0.016	Hydrogen site location: inferred from neighbouring sites
*wR*(*F*^2^) = 0.071	H-atom parameters constrained
*S* = 1.39	*w* = 1/[σ^2^(*F*_o_^2^) + (0.039*P*)^2^ + 0.2397*P*] where *P* = (*F*_o_^2^ + 2*F*_c_^2^)/3
11933 reflections	(Δ/σ)_max_ < 0.001
379 parameters	Δρ_max_ = 1.25 e Å^−^^3^
0 restraints	Δρ_min_ = −1.51 e Å^−^^3^

### Special details

**Table:**

Experimental. The crystal was placed in the cold stream of an Oxford Cyrosystems Cobra open-flow nitrogen cryostat (Cosier & Glazer, 1986) operating at 100.0 (1)K.
Geometry. All esds (except the esd in the dihedral angle between two l.s. planes) are estimated using the full covariance matrix. The cell esds are taken into account individually in the estimation of esds in distances, angles and torsion angles; correlations between esds in cell parameters are only used when they are defined by crystal symmetry. An approximate (isotropic) treatment of cell esds is used for estimating esds involving l.s. planes.
Refinement. Refinement of F^2^ against ALL reflections. The weighted R-factor wR and goodness of fit S are based on F^2^, conventional R-factors R are based on F, with F set to zero for negative F^2^. The threshold expression of F^2^ > 2sigma(F^2^) is used only for calculating R-factors(gt) etc. and is not relevant to the choice of reflections for refinement. R-factors based on F^2^ are statistically about twice as large as those based on F, and R- factors based on ALL data will be even larger.

### Fractional atomic coordinates and isotropic or equivalent isotropic displacement parameters (Å^2^)

**Table:**

	*x*	*y*	*z*	*U*_iso_*/*U*_eq_	
Nd1	0.135991 (5)	0.561522 (5)	0.646953 (5)	0.01014 (3)	
O1	0.07381 (10)	0.68363 (9)	0.82160 (9)	0.01641 (16)	
O2	0.27604 (10)	0.77974 (10)	0.77735 (9)	0.01605 (16)	
O3	0.15987 (11)	0.35844 (10)	0.56526 (9)	0.01848 (18)	
O4	0.00549 (10)	0.36146 (9)	0.44049 (9)	0.01530 (16)	
O5	0.08829 (10)	0.59147 (10)	0.34526 (9)	0.01613 (16)	
O6	0.24489 (10)	0.63755 (10)	0.49909 (9)	0.01578 (16)	
N1	0.19179 (12)	0.48650 (11)	0.82052 (10)	0.01452 (17)	
N2	0.38701 (11)	0.57086 (11)	0.69470 (10)	0.01398 (17)	
C1	0.30009 (13)	0.45576 (12)	0.82829 (11)	0.01378 (19)	
C2	0.10022 (15)	0.45161 (13)	0.88670 (12)	0.0171 (2)	
H2A	0.0266	0.4735	0.8822	0.021*	
C3	0.10869 (16)	0.38359 (14)	0.96296 (12)	0.0192 (2)	
H3A	0.0440	0.3636	1.0096	0.023*	
C4	0.21506 (16)	0.34718 (14)	0.96717 (12)	0.0195 (2)	
H4A	0.2211	0.2992	1.0148	0.023*	
C5	0.31456 (14)	0.38298 (13)	0.89895 (12)	0.0166 (2)	
C6	0.42959 (16)	0.35029 (14)	0.90061 (13)	0.0202 (2)	
H6A	0.4371	0.2993	0.9446	0.024*	
C7	0.52686 (16)	0.39300 (15)	0.83879 (13)	0.0203 (2)	
H7A	0.6007	0.3712	0.8412	0.024*	
C8	0.51832 (13)	0.47134 (13)	0.76955 (12)	0.0163 (2)	
C9	0.61982 (14)	0.52029 (14)	0.70660 (13)	0.0191 (2)	
H9A	0.6982	0.5055	0.7114	0.023*	
C10	0.60218 (14)	0.59012 (14)	0.63790 (13)	0.0192 (2)	
H10A	0.6673	0.6218	0.5946	0.023*	
C11	0.48338 (14)	0.61251 (13)	0.63463 (12)	0.0169 (2)	
H11A	0.4718	0.6592	0.5876	0.020*	
C12	0.40383 (13)	0.50067 (12)	0.76196 (11)	0.01365 (19)	
C13	0.18596 (13)	0.78037 (12)	0.83975 (11)	0.01332 (19)	
C14	0.21680 (13)	0.89994 (12)	0.93665 (10)	0.01395 (19)	
C15	0.11566 (16)	0.91061 (14)	0.99806 (12)	0.0187 (2)	
H15A	0.0276	0.8449	0.9768	0.022*	
C16	0.14606 (19)	1.01942 (16)	1.09120 (13)	0.0238 (3)	
H16A	0.0783	1.0268	1.1317	0.029*	
C17	0.2775 (2)	1.11663 (16)	1.12335 (13)	0.0259 (3)	
H17A	0.2983	1.1883	1.1866	0.031*	
C18	0.37869 (17)	1.10783 (14)	1.06176 (13)	0.0231 (3)	
H18A	0.4667	1.1736	1.0832	0.028*	
C19	0.34719 (15)	0.99948 (13)	0.96739 (12)	0.0176 (2)	
H19A	0.4140	0.9940	0.9249	0.021*	
C20	0.06862 (12)	0.30308 (12)	0.47633 (11)	0.01305 (18)	
C21	0.03417 (13)	0.16578 (11)	0.41377 (11)	0.01327 (18)	
C22	0.12719 (14)	0.11477 (13)	0.43391 (12)	0.0163 (2)	
H22A	0.2112	0.1674	0.4826	0.020*	
C23	0.09434 (16)	−0.01436 (14)	0.38135 (13)	0.0196 (2)	
H23A	0.1570	−0.0478	0.3939	0.023*	
C24	−0.03202 (17)	−0.09346 (14)	0.31012 (13)	0.0214 (2)	
H24A	−0.0544	−0.1802	0.2758	0.026*	
C25	−0.12551 (16)	−0.04316 (13)	0.28996 (13)	0.0208 (2)	
H25A	−0.2102	−0.0963	0.2424	0.025*	
C26	−0.09184 (14)	0.08677 (13)	0.34117 (12)	0.0163 (2)	
H26A	−0.1535	0.1207	0.3269	0.020*	
C27	0.20609 (13)	0.65426 (12)	0.40696 (11)	0.01327 (19)	
C28	0.30895 (12)	0.75547 (12)	0.36677 (11)	0.01323 (18)	
C29	0.26872 (14)	0.78761 (13)	0.27284 (12)	0.0171 (2)	
H29A	0.1782	0.7453	0.2342	0.020*	
C30	0.36372 (16)	0.88293 (15)	0.23681 (14)	0.0211 (2)	
H30A	0.3362	0.9050	0.1750	0.025*	
C31	0.49950 (16)	0.94497 (14)	0.29314 (14)	0.0218 (2)	
H31A	0.5628	1.0087	0.2692	0.026*	
C32	0.54037 (15)	0.91166 (15)	0.38495 (14)	0.0230 (3)	
H32A	0.6316	0.9519	0.4217	0.028*	
C33	0.44521 (14)	0.81793 (14)	0.42247 (12)	0.0192 (2)	
H33A	0.4729	0.7970	0.4850	0.023*	

### Atomic displacement parameters (Å^2^)

**Table:**

	*U*^11^	*U*^22^	*U*^33^	*U*^12^	*U*^13^	*U*^23^
Nd1	0.01005 (3)	0.00866 (3)	0.01053 (3)	0.00329 (2)	0.00192 (2)	0.00210 (2)
O1	0.0166 (4)	0.0123 (4)	0.0174 (4)	0.0039 (3)	0.0051 (3)	0.0028 (3)
O2	0.0153 (4)	0.0136 (4)	0.0154 (4)	0.0041 (3)	0.0038 (3)	0.0012 (3)
O3	0.0172 (4)	0.0130 (4)	0.0209 (4)	0.0069 (3)	−0.0032 (3)	−0.0010 (3)
O4	0.0173 (4)	0.0138 (4)	0.0179 (4)	0.0090 (3)	0.0041 (3)	0.0057 (3)
O5	0.0132 (4)	0.0154 (4)	0.0163 (4)	0.0025 (3)	0.0025 (3)	0.0049 (3)
O6	0.0148 (4)	0.0176 (4)	0.0156 (4)	0.0058 (3)	0.0044 (3)	0.0074 (3)
N1	0.0149 (4)	0.0144 (4)	0.0145 (4)	0.0065 (3)	0.0031 (3)	0.0042 (3)
N2	0.0132 (4)	0.0125 (4)	0.0138 (4)	0.0044 (3)	0.0016 (3)	0.0021 (3)
C1	0.0148 (5)	0.0123 (4)	0.0127 (4)	0.0058 (4)	0.0007 (4)	0.0017 (4)
C2	0.0194 (5)	0.0165 (5)	0.0168 (5)	0.0079 (4)	0.0058 (4)	0.0060 (4)
C3	0.0229 (6)	0.0189 (6)	0.0176 (5)	0.0087 (5)	0.0066 (5)	0.0078 (4)
C4	0.0241 (6)	0.0177 (5)	0.0174 (5)	0.0084 (5)	0.0032 (4)	0.0072 (4)
C5	0.0185 (5)	0.0147 (5)	0.0157 (5)	0.0073 (4)	0.0000 (4)	0.0037 (4)
C6	0.0236 (6)	0.0174 (5)	0.0209 (6)	0.0109 (5)	−0.0003 (5)	0.0054 (4)
C7	0.0210 (6)	0.0189 (6)	0.0219 (6)	0.0125 (5)	−0.0003 (5)	0.0023 (5)
C8	0.0154 (5)	0.0145 (5)	0.0172 (5)	0.0078 (4)	0.0007 (4)	0.0004 (4)
C9	0.0161 (5)	0.0173 (5)	0.0221 (6)	0.0087 (4)	0.0036 (4)	0.0005 (4)
C10	0.0153 (5)	0.0185 (5)	0.0215 (6)	0.0067 (4)	0.0057 (4)	0.0023 (4)
C11	0.0146 (5)	0.0162 (5)	0.0178 (5)	0.0050 (4)	0.0037 (4)	0.0040 (4)
C12	0.0131 (4)	0.0120 (4)	0.0135 (5)	0.0053 (4)	0.0005 (4)	0.0005 (4)
C13	0.0151 (5)	0.0119 (4)	0.0124 (4)	0.0057 (4)	0.0020 (4)	0.0031 (4)
C14	0.0177 (5)	0.0122 (4)	0.0114 (4)	0.0067 (4)	0.0013 (4)	0.0026 (4)
C15	0.0237 (6)	0.0174 (5)	0.0171 (5)	0.0105 (5)	0.0060 (4)	0.0051 (4)
C16	0.0362 (8)	0.0233 (6)	0.0171 (6)	0.0191 (6)	0.0069 (5)	0.0037 (5)
C17	0.0409 (9)	0.0196 (6)	0.0164 (6)	0.0175 (6)	−0.0023 (5)	−0.0014 (5)
C18	0.0286 (7)	0.0146 (5)	0.0190 (6)	0.0065 (5)	−0.0049 (5)	0.0002 (4)
C19	0.0204 (5)	0.0142 (5)	0.0150 (5)	0.0058 (4)	−0.0009 (4)	0.0025 (4)
C20	0.0128 (4)	0.0101 (4)	0.0160 (5)	0.0053 (4)	0.0034 (4)	0.0027 (4)
C21	0.0143 (5)	0.0105 (4)	0.0146 (5)	0.0056 (4)	0.0024 (4)	0.0026 (4)
C22	0.0175 (5)	0.0141 (5)	0.0179 (5)	0.0087 (4)	0.0025 (4)	0.0030 (4)
C23	0.0259 (6)	0.0153 (5)	0.0200 (6)	0.0125 (5)	0.0047 (5)	0.0033 (4)
C24	0.0308 (7)	0.0131 (5)	0.0185 (5)	0.0099 (5)	0.0026 (5)	0.0011 (4)
C25	0.0235 (6)	0.0131 (5)	0.0192 (6)	0.0045 (4)	−0.0017 (5)	0.0008 (4)
C26	0.0173 (5)	0.0126 (5)	0.0165 (5)	0.0051 (4)	−0.0003 (4)	0.0033 (4)
C27	0.0137 (5)	0.0115 (4)	0.0145 (5)	0.0050 (4)	0.0047 (4)	0.0037 (4)
C28	0.0132 (4)	0.0116 (4)	0.0148 (5)	0.0045 (4)	0.0048 (4)	0.0044 (4)
C29	0.0158 (5)	0.0173 (5)	0.0211 (5)	0.0076 (4)	0.0048 (4)	0.0096 (4)
C30	0.0224 (6)	0.0202 (6)	0.0251 (6)	0.0089 (5)	0.0073 (5)	0.0136 (5)
C31	0.0216 (6)	0.0178 (6)	0.0246 (6)	0.0039 (5)	0.0084 (5)	0.0104 (5)
C32	0.0170 (5)	0.0219 (6)	0.0228 (6)	−0.0005 (5)	0.0024 (5)	0.0093 (5)
C33	0.0153 (5)	0.0191 (5)	0.0189 (5)	0.0016 (4)	0.0020 (4)	0.0082 (4)

### Geometric parameters (Å, °)

**Table:**

Nd1—O4^i^	2.3856 (10)	C10—H10A	0.9300
Nd1—O6	2.4060 (10)	C11—H11A	0.9300
Nd1—O5^i^	2.4230 (10)	C13—C14	1.4996 (18)
Nd1—O2	2.4600 (10)	C14—C19	1.3879 (19)
Nd1—O3	2.4810 (10)	C14—C15	1.3942 (19)
Nd1—O1	2.5475 (10)	C15—C16	1.393 (2)
Nd1—N1	2.6288 (12)	C15—H15A	0.9300
Nd1—N2	2.6870 (11)	C16—C17	1.385 (3)
Nd1—O4	2.8039 (10)	C16—H16A	0.9300
O1—C13	1.2591 (16)	C17—C18	1.390 (3)
O2—C13	1.2781 (16)	C17—H17A	0.9300
O3—C20	1.2553 (16)	C18—C19	1.396 (2)
O4—C20	1.2736 (15)	C18—H18A	0.9300
O4—Nd1^i^	2.3855 (10)	C19—H19A	0.9300
O5—C27	1.2607 (16)	C20—C21	1.4932 (17)
O5—Nd1^i^	2.4230 (10)	C21—C26	1.3925 (18)
O6—C27	1.2684 (16)	C21—C22	1.3974 (18)
N1—C2	1.3304 (17)	C22—C23	1.3887 (19)
N1—C1	1.3579 (17)	C22—H22A	0.9300
N2—C11	1.3266 (17)	C23—C24	1.389 (2)
N2—C12	1.3636 (17)	C23—H23A	0.9300
C1—C5	1.4122 (19)	C24—C25	1.395 (2)
C1—C12	1.4427 (18)	C24—H24A	0.9300
C2—C3	1.404 (2)	C25—C26	1.3929 (19)
C2—H2A	0.9300	C25—H25A	0.9300
C3—C4	1.378 (2)	C26—H26A	0.9300
C3—H3A	0.9300	C27—C28	1.5034 (17)
C4—C5	1.408 (2)	C28—C33	1.3918 (19)
C4—H4A	0.9300	C28—C29	1.3965 (19)
C5—C6	1.438 (2)	C29—C30	1.3939 (19)
C6—C7	1.351 (2)	C29—H29A	0.9300
C6—H6A	0.9300	C30—C31	1.390 (2)
C7—C8	1.435 (2)	C30—H30A	0.9300
C7—H7A	0.9300	C31—C32	1.385 (2)
C8—C9	1.408 (2)	C31—H31A	0.9300
C8—C12	1.4118 (18)	C32—C33	1.394 (2)
C9—C10	1.376 (2)	C32—H32A	0.9300
C9—H9A	0.9300	C33—H33A	0.9300
C10—C11	1.407 (2)		
			
O4^i^—Nd1—O6	73.10 (3)	C9—C10—H10A	120.7
O4^i^—Nd1—O5^i^	79.74 (3)	C11—C10—H10A	120.7
O6—Nd1—O5^i^	135.40 (3)	N2—C11—C10	123.81 (13)
O4^i^—Nd1—O2	90.44 (4)	N2—C11—H11A	118.1
O6—Nd1—O2	86.17 (3)	C10—C11—H11A	118.1
O5^i^—Nd1—O2	129.27 (3)	N2—C12—C8	122.72 (12)
O4^i^—Nd1—O3	126.97 (3)	N2—C12—C1	118.10 (11)
O6—Nd1—O3	88.34 (4)	C8—C12—C1	119.17 (12)
O5^i^—Nd1—O3	80.45 (4)	O1—C13—O2	121.55 (12)
O2—Nd1—O3	138.41 (3)	O1—C13—C14	120.10 (11)
O4^i^—Nd1—O1	78.44 (3)	O2—C13—C14	118.35 (11)
O6—Nd1—O1	129.08 (3)	O1—C13—Nd1	62.75 (7)
O5^i^—Nd1—O1	76.84 (3)	O2—C13—Nd1	58.84 (7)
O2—Nd1—O1	52.45 (3)	C14—C13—Nd1	176.57 (9)
O3—Nd1—O1	141.78 (4)	C19—C14—C15	119.60 (12)
O4^i^—Nd1—N1	146.80 (3)	C19—C14—C13	120.12 (12)
O6—Nd1—N1	138.90 (3)	C15—C14—C13	120.26 (12)
O5^i^—Nd1—N1	78.15 (3)	C16—C15—C14	120.19 (14)
O2—Nd1—N1	84.74 (4)	C16—C15—H15A	119.9
O3—Nd1—N1	72.90 (4)	C14—C15—H15A	119.9
O1—Nd1—N1	72.63 (3)	C17—C16—C15	119.79 (15)
O4^i^—Nd1—N2	147.67 (3)	C17—C16—H16A	120.1
O6—Nd1—N2	77.31 (3)	C15—C16—H16A	120.1
O5^i^—Nd1—N2	131.81 (3)	C16—C17—C18	120.50 (14)
O2—Nd1—N2	74.72 (3)	C16—C17—H17A	119.8
O3—Nd1—N2	63.86 (3)	C18—C17—H17A	119.8
O1—Nd1—N2	111.81 (3)	C17—C18—C19	119.49 (15)
N1—Nd1—N2	61.62 (3)	C17—C18—H18A	120.3
O4^i^—Nd1—O4	78.19 (3)	C19—C18—H18A	120.3
O6—Nd1—O4	73.81 (3)	C14—C19—C18	120.38 (14)
O5^i^—Nd1—O4	66.35 (3)	C14—C19—H19A	119.8
O2—Nd1—O4	159.09 (3)	C18—C19—H19A	119.8
O3—Nd1—O4	48.81 (3)	O3—C20—O4	121.21 (12)
O1—Nd1—O4	139.16 (3)	O3—C20—C21	118.34 (11)
N1—Nd1—O4	114.30 (3)	O4—C20—C21	120.45 (11)
N2—Nd1—O4	105.85 (3)	O3—C20—Nd1	53.53 (6)
C13—O1—Nd1	91.18 (8)	O4—C20—Nd1	68.32 (7)
C13—O2—Nd1	94.76 (8)	C21—C20—Nd1	167.86 (9)
C20—O3—Nd1	102.46 (8)	C26—C21—C22	119.87 (12)
C20—O4—Nd1^i^	171.01 (9)	C26—C21—C20	120.95 (11)
C20—O4—Nd1	86.72 (7)	C22—C21—C20	119.08 (11)
Nd1^i^—O4—Nd1	101.81 (3)	C23—C22—C21	120.12 (13)
C27—O5—Nd1^i^	139.15 (9)	C23—C22—H22A	119.9
C27—O6—Nd1	135.75 (8)	C21—C22—H22A	119.9
C2—N1—C1	118.06 (12)	C24—C23—C22	119.98 (13)
C2—N1—Nd1	120.55 (9)	C24—C23—H23A	120.0
C1—N1—Nd1	119.81 (8)	C22—C23—H23A	120.0
C11—N2—C12	117.69 (12)	C23—C24—C25	120.16 (13)
C11—N2—Nd1	122.51 (9)	C23—C24—H24A	119.9
C12—N2—Nd1	117.62 (8)	C25—C24—H24A	119.9
N1—C1—C5	122.46 (12)	C26—C25—C24	119.92 (13)
N1—C1—C12	118.03 (11)	C26—C25—H25A	120.0
C5—C1—C12	119.51 (12)	C24—C25—H25A	120.0
N1—C2—C3	123.59 (13)	C25—C26—C21	119.94 (13)
N1—C2—H2A	118.2	C25—C26—H26A	120.0
C3—C2—H2A	118.2	C21—C26—H26A	120.0
C4—C3—C2	118.44 (13)	O5—C27—O6	125.19 (12)
C4—C3—H3A	120.8	O5—C27—C28	117.15 (11)
C2—C3—H3A	120.8	O6—C27—C28	117.66 (11)
C3—C4—C5	119.63 (13)	C33—C28—C29	119.27 (12)
C3—C4—H4A	120.2	C33—C28—C27	120.46 (12)
C5—C4—H4A	120.2	C29—C28—C27	120.27 (11)
C4—C5—C1	117.69 (12)	C30—C29—C28	120.22 (13)
C4—C5—C6	122.59 (13)	C30—C29—H29A	119.9
C1—C5—C6	119.70 (13)	C28—C29—H29A	119.9
C7—C6—C5	120.58 (13)	C31—C30—C29	120.09 (13)
C7—C6—H6A	119.7	C31—C30—H30A	120.0
C5—C6—H6A	119.7	C29—C30—H30A	120.0
C6—C7—C8	121.22 (13)	C32—C31—C30	119.87 (13)
C6—C7—H7A	119.4	C32—C31—H31A	120.1
C8—C7—H7A	119.4	C30—C31—H31A	120.1
C9—C8—C12	117.66 (13)	C31—C32—C33	120.20 (14)
C9—C8—C7	122.62 (13)	C31—C32—H32A	119.9
C12—C8—C7	119.71 (13)	C33—C32—H32A	119.9
C10—C9—C8	119.56 (13)	C28—C33—C32	120.33 (13)
C10—C9—H9A	120.2	C28—C33—H33A	119.8
C8—C9—H9A	120.2	C32—C33—H33A	119.8
C9—C10—C11	118.52 (13)		
			
O4^i^—Nd1—O1—C13	−98.30 (8)	O1—Nd1—N2—C12	72.08 (9)
O6—Nd1—O1—C13	−41.56 (9)	N1—Nd1—N2—C12	17.66 (8)
O5^i^—Nd1—O1—C13	179.68 (8)	O4—Nd1—N2—C12	−91.79 (9)
O2—Nd1—O1—C13	1.33 (7)	C13—Nd1—N2—C12	91.68 (9)
O3—Nd1—O1—C13	124.60 (8)	C20—Nd1—N2—C12	−77.39 (9)
N1—Nd1—O1—C13	98.18 (8)	Nd1^i^—Nd1—N2—C12	−117.61 (8)
N2—Nd1—O1—C13	49.60 (8)	C2—N1—C1—C5	3.41 (19)
O4—Nd1—O1—C13	−154.52 (7)	Nd1—N1—C1—C5	−162.33 (10)
C20—Nd1—O1—C13	165.30 (8)	C2—N1—C1—C12	−176.14 (12)
Nd1^i^—Nd1—O1—C13	−122.72 (7)	Nd1—N1—C1—C12	18.12 (15)
O4^i^—Nd1—O2—C13	73.69 (8)	C1—N1—C2—C3	−0.8 (2)
O6—Nd1—O2—C13	146.72 (8)	Nd1—N1—C2—C3	164.86 (11)
O5^i^—Nd1—O2—C13	−3.38 (10)	N1—C2—C3—C4	−2.1 (2)
O3—Nd1—O2—C13	−130.13 (8)	C2—C3—C4—C5	2.4 (2)
O1—Nd1—O2—C13	−1.31 (7)	C3—C4—C5—C1	0.0 (2)
N1—Nd1—O2—C13	−73.41 (8)	C3—C4—C5—C6	178.85 (14)
N2—Nd1—O2—C13	−135.39 (8)	N1—C1—C5—C4	−3.0 (2)
O4—Nd1—O2—C13	130.12 (9)	C12—C1—C5—C4	176.50 (12)
C20—Nd1—O2—C13	−155.92 (10)	N1—C1—C5—C6	178.08 (12)
Nd1^i^—Nd1—O2—C13	89.53 (8)	C12—C1—C5—C6	−2.39 (19)
O4^i^—Nd1—O3—C20	−7.61 (10)	C4—C5—C6—C7	−176.12 (14)
O6—Nd1—O3—C20	−74.92 (9)	C1—C5—C6—C7	2.7 (2)
O5^i^—Nd1—O3—C20	61.73 (9)	C5—C6—C7—C8	−0.3 (2)
O2—Nd1—O3—C20	−157.25 (8)	C6—C7—C8—C9	178.22 (14)
O1—Nd1—O3—C20	115.79 (9)	C6—C7—C8—C12	−2.5 (2)
N1—Nd1—O3—C20	142.18 (9)	C12—C8—C9—C10	−2.2 (2)
N2—Nd1—O3—C20	−151.59 (10)	C7—C8—C9—C10	177.14 (14)
O4—Nd1—O3—C20	−5.11 (7)	C8—C9—C10—C11	1.2 (2)
C13—Nd1—O3—C20	160.95 (8)	C12—N2—C11—C10	−1.04 (19)
Nd1^i^—Nd1—O3—C20	−6.30 (8)	Nd1—N2—C11—C10	−163.83 (10)
O4^i^—Nd1—O4—C20	−177.11 (9)	C9—C10—C11—N2	0.5 (2)
O6—Nd1—O4—C20	107.28 (8)	C11—N2—C12—C8	−0.05 (18)
O5^i^—Nd1—O4—C20	−93.26 (8)	Nd1—N2—C12—C8	163.59 (9)
O2—Nd1—O4—C20	124.56 (10)	C11—N2—C12—C1	179.54 (11)
O3—Nd1—O4—C20	4.93 (7)	Nd1—N2—C12—C1	−16.82 (14)
O1—Nd1—O4—C20	−120.82 (8)	C9—C8—C12—N2	1.64 (19)
N1—Nd1—O4—C20	−29.59 (8)	C7—C8—C12—N2	−177.71 (12)
N2—Nd1—O4—C20	35.95 (8)	C9—C8—C12—C1	−177.94 (12)
C13—Nd1—O4—C20	−154.15 (10)	C7—C8—C12—C1	2.71 (18)
Nd1^i^—Nd1—O4—C20	−177.11 (9)	N1—C1—C12—N2	−0.35 (17)
O4^i^—Nd1—O4—Nd1^i^	0.0	C5—C1—C12—N2	−179.91 (12)
O6—Nd1—O4—Nd1^i^	−75.61 (4)	N1—C1—C12—C8	179.25 (11)
O5^i^—Nd1—O4—Nd1^i^	83.85 (4)	C5—C1—C12—C8	−0.30 (18)
O2—Nd1—O4—Nd1^i^	−58.33 (10)	Nd1—O1—C13—O2	−2.37 (13)
O3—Nd1—O4—Nd1^i^	−177.96 (6)	Nd1—O1—C13—C14	177.83 (10)
O1—Nd1—O4—Nd1^i^	56.29 (6)	Nd1—O2—C13—O1	2.47 (14)
N1—Nd1—O4—Nd1^i^	147.52 (3)	Nd1—O2—C13—C14	−177.74 (10)
N2—Nd1—O4—Nd1^i^	−146.94 (3)	O1—C13—C14—C19	170.57 (13)
C13—Nd1—O4—Nd1^i^	22.96 (11)	O2—C13—C14—C19	−9.23 (19)
C20—Nd1—O4—Nd1^i^	177.11 (9)	Nd1—C13—C14—C19	−43.6 (16)
O4^i^—Nd1—O6—C27	−24.01 (12)	O1—C13—C14—C15	−7.96 (19)
O5^i^—Nd1—O6—C27	31.03 (14)	O2—C13—C14—C15	172.24 (12)
O2—Nd1—O6—C27	−115.64 (13)	Nd1—C13—C14—C15	137.8 (15)
O3—Nd1—O6—C27	105.61 (12)	C19—C14—C15—C16	−1.3 (2)
O1—Nd1—O6—C27	−82.91 (13)	C13—C14—C15—C16	177.23 (13)
N1—Nd1—O6—C27	166.87 (11)	C14—C15—C16—C17	−0.6 (2)
N2—Nd1—O6—C27	169.17 (13)	C15—C16—C17—C18	1.5 (2)
O4—Nd1—O6—C27	58.26 (12)	C16—C17—C18—C19	−0.5 (2)
C13—Nd1—O6—C27	−100.77 (12)	C15—C14—C19—C18	2.3 (2)
C20—Nd1—O6—C27	82.26 (12)	C13—C14—C19—C18	−176.25 (13)
Nd1^i^—Nd1—O6—C27	21.25 (12)	C17—C18—C19—C14	−1.4 (2)
O4^i^—Nd1—N1—C2	18.15 (14)	Nd1—O3—C20—O4	9.94 (14)
O6—Nd1—N1—C2	178.90 (9)	Nd1—O3—C20—C21	−169.30 (9)
O5^i^—Nd1—N1—C2	−31.09 (10)	Nd1^i^—O4—C20—O3	−170.2 (5)
O2—Nd1—N1—C2	100.88 (10)	Nd1—O4—C20—O3	−8.59 (12)
O3—Nd1—N1—C2	−114.61 (11)	Nd1^i^—O4—C20—C21	9.0 (6)
O1—Nd1—N1—C2	48.65 (10)	Nd1—O4—C20—C21	170.64 (11)
N2—Nd1—N1—C2	176.34 (11)	Nd1^i^—O4—C20—Nd1	−161.6 (6)
O4—Nd1—N1—C2	−88.11 (10)	O3—C20—C21—C26	157.87 (13)
C13—Nd1—N1—C2	75.05 (10)	O4—C20—C21—C26	−21.38 (19)
C20—Nd1—N1—C2	−100.15 (10)	Nd1—C20—C21—C26	112.6 (4)
Nd1^i^—Nd1—N1—C2	−58.31 (12)	O3—C20—C21—C22	−18.49 (18)
O4^i^—Nd1—N1—C1	−176.47 (8)	O4—C20—C21—C22	162.26 (12)
O6—Nd1—N1—C1	−15.72 (12)	Nd1—C20—C21—C22	−63.7 (4)
O5^i^—Nd1—N1—C1	134.29 (10)	C26—C21—C22—C23	0.2 (2)
O2—Nd1—N1—C1	−93.74 (10)	C20—C21—C22—C23	176.63 (12)
O3—Nd1—N1—C1	50.77 (9)	C21—C22—C23—C24	−1.0 (2)
O1—Nd1—N1—C1	−145.97 (10)	C22—C23—C24—C25	0.9 (2)
N2—Nd1—N1—C1	−18.27 (9)	C23—C24—C25—C26	0.2 (2)
O4—Nd1—N1—C1	77.27 (10)	C24—C25—C26—C21	−1.0 (2)
C13—Nd1—N1—C1	−119.57 (10)	C22—C21—C26—C25	0.8 (2)
C20—Nd1—N1—C1	65.23 (10)	C20—C21—C26—C25	−175.54 (13)
Nd1^i^—Nd1—N1—C1	107.07 (9)	Nd1^i^—O5—C27—O6	10.3 (2)
O4^i^—Nd1—N2—C11	−21.91 (13)	Nd1^i^—O5—C27—C28	−170.72 (9)
O6—Nd1—N2—C11	2.17 (10)	Nd1—O6—C27—O5	−28.0 (2)
O5^i^—Nd1—N2—C11	143.22 (9)	Nd1—O6—C27—C28	152.96 (9)
O2—Nd1—N2—C11	−87.28 (10)	O5—C27—C28—C33	−172.26 (13)
O3—Nd1—N2—C11	96.61 (11)	O6—C27—C28—C33	6.83 (18)
O1—Nd1—N2—C11	−125.12 (10)	O5—C27—C28—C29	7.60 (18)
N1—Nd1—N2—C11	−179.55 (11)	O6—C27—C28—C29	−173.31 (12)
O4—Nd1—N2—C11	71.01 (10)	C33—C28—C29—C30	−1.2 (2)
C13—Nd1—N2—C11	−105.52 (10)	C27—C28—C29—C30	178.96 (13)
C20—Nd1—N2—C11	85.41 (10)	C28—C29—C30—C31	1.0 (2)
Nd1^i^—Nd1—N2—C11	45.18 (11)	C29—C30—C31—C32	0.2 (2)
O4^i^—Nd1—N2—C12	175.30 (8)	C30—C31—C32—C33	−1.3 (3)
O6—Nd1—N2—C12	−160.63 (9)	C29—C28—C33—C32	0.1 (2)
O5^i^—Nd1—N2—C12	−19.57 (11)	C27—C28—C33—C32	179.95 (14)
O2—Nd1—N2—C12	109.92 (9)	C31—C32—C33—C28	1.1 (2)
O3—Nd1—N2—C12	−66.19 (9)		

Symmetry codes: (i) −*x*, −*y*+1, −*z*+1.

### Hydrogen-bond geometry (Å, °)

**Table:**

Cg1, Cg2 and Cg5 are the centroids of the C28–C33, C21–C26 and C14–C19 phenyl rings, respectively. Cg3 and Cg4 are the centroids of the N2/C8–C12 and N1/C1–C15 pyridine rings, respectively.

**Table:**

*D*—H···*A*	*D*—H	H···*A*	*D*···*A*	*D*—H···*A*
C3—H3A···O1^ii^	0.93	2.57	3.4729 (19)	163
C11—H11A···O6	0.93	2.50	3.1239 (19)	125
C26—H26A···O2^i^	0.93	2.56	3.4393 (19)	158
C7—H7A···Cg1^iii^	0.93	2.89	3.4554 (18)	121
C16—H16A···Cg2^iv^	0.93	2.98	3.7544 (18)	141
C17—H17A···Cg3^v^	0.93	2.94	3.7287 (19)	143
C24—H24A···Cg4^vi^	0.93	2.81	3.6620 (17)	153
C30—H30A···Cg5^vii^	0.93	2.73	3.6435 (18)	167

Symmetry codes: (ii) −*x*, −*y*+1, −*z*+2; (i) −*x*, −*y*+1, −*z*+1; (iii) −*x*+1, −*y*+1, −*z*+1; (iv) *x*, *y*+1, *z*+1; (v) −*x*+1, −*y*+2, −*z*+2; (vi) −*x*, −*y*, −*z*+1; (vii) *x*, *y*, *z*−1.
